# Assembly and phylogenetic analysis of the complete chloroplast genome of *Citrus aurantium* (Rutaceae)

**DOI:** 10.1080/23802359.2020.1771228

**Published:** 2020-06-01

**Authors:** Haifeng Lin, Xinyi Li, Di Bai

**Affiliations:** a College of Information Science and Technology, Nanjing Forestry University, Nanjing, Jiangsu, China;; b College of Art and Design, Nanjing Forestry University, Nanjing, Jiangsu, China;; c College of Engineering, Nanjing Agricultural University, Nanjing, Jiangsu, China

**Keywords:** Rutaceae, *Citrus aurantium*, chloroplast genome, phylogenetic analysis

## Abstract

*Citrus aurantium* (*C. aurantium*), belonging to the family Rutaceae, is usually utilized as a flavoring and acidifying agent for food. This study assembled and characterized the complete chloroplast (cp) genome of *C. aurantium*. The cp genome was 160,140 bp in length, containing a pair of inverted repeats (IRs, 26,996 bp each), which is separated by a large single-copy (LSC, 87,763 bp) region and a small single copy (SSC, 18,385 bp) region. The cp genome has overall GC content of 38.48% and 135 genes, composing of 90 protein-coding genes, 37 tRNA genes and 8 rRNA genes. Phylogenetic analysis based on 25 cp genomes highly supported that *C. aurantium* was evolutionarily close to *Cirtus sinensis* (*C. sinensis*).

*Citrus aurantium*, also known as bitter orange, is a flowering, fruit-bearing evergreen tree that belongs to the family Rutaceae. The origin of *C. aurantium* is south east Asia, and it has been spread by human to many parts of the world. The essential oil of bitter orange is popularly used as a flavoring or solvent and also for consumption. Additionally, bitter orange is also employed in herbal medicine as a stimulant and appetite suppressant, due to its active ingredient, synephrine (Sharpe et al. 2006). However, the bitter orange supplements may be linked to a number of serious side effects and deaths, and it is still not concluded if bitter orange affects medical conditions of heart and cardiovascular organs (Carvalho-Freitas and Costa 2002; Fugh-Berman and Myers 2004). In this study, we assembled and characterized the complete cp genome of *C. aurantium*, which would provide basic genetic resource for the research of its various applications.

The genomic DNA was extracted from the fresh leaves of *C. aurantium* collected from Citrus Research Institute of CAAS, Chongqing, China (29°45′36.2″N, 106°22′40.5″E). The voucher specimen is now deposited at the Herbarium of Nanjing Forestry University (accession number: 20190526JJDD01). Purified DNA was then fragmented to construct an Illumina paired-end library, and then sequenced using the Illumina NovaSeq 6000 platform. The raw sequencing data were filtered and trimmed by fastp program (Chen et al. 2018), and then fed into NOVOPlasty version 3.7.2 (Dierckxsens et al. 2017) for assembly using the *psbA* gene and the complete cp genome sequence of *C. sinensis* (GenBank accession: NC_008334.1) as the seed and reference genome, respectively. The assembled genome was then annotated using PGA (Qu et al. 2019) against the cp genome of *C. sinensis* and adjusted manually as needed using Macvector v17.0.7. The complete cp genome was submitted to GenBank (accession number: MT106672).

The cp genome of *C. aurantium* was 160,140 bp (GC content: 38.48%) in length, composing of a LSC region of 87,763 bp (GC content: 36.81%) and a SSC region of 18,385 bp (GC content: 33.38%) separated by a pair of 26,996 bp IR regions (GC content: 42.93%). The *C. aurantium* cp genome encoded a total of 135 genes, including 90 protein-coding genes, 37 tRNA genes and 8 rRNA genes. Additionally, twelve protein-coding genes (*rps16*, *atpF*, *rpoC1*, *rps12*×2, *petB*, *petD*, *rpl2*×2, *ndhB* × 2, and *ndhA*) and 8 tRNA genes (*trnK-UUU*, *trnG-GCC*, *trnL-UAA*, *trnV-UAC*, *trnI-GAU* × 2, and *trnA-UGC* × 2) were found to contain one intron, and two genes contain two introns (*ycf3* and *clpP*). Phylogenetic analysis was carried out using 75 conserved protein-coding genes with those of 25 plant cp genomes by Neighbor-Joining (NJ) method in MEGA X (Kumar et al. 2018). The phylogenetic tree highly supported that *C. aurantium* was clustered to another species in the Rutaceae family (*C. sinensis*) with 100% bootstrap values (Figure 1). The complete cp genome of *C, aurantium* will provide a useful basic genetic resource for the research of its various applications.

**Figure 1.**
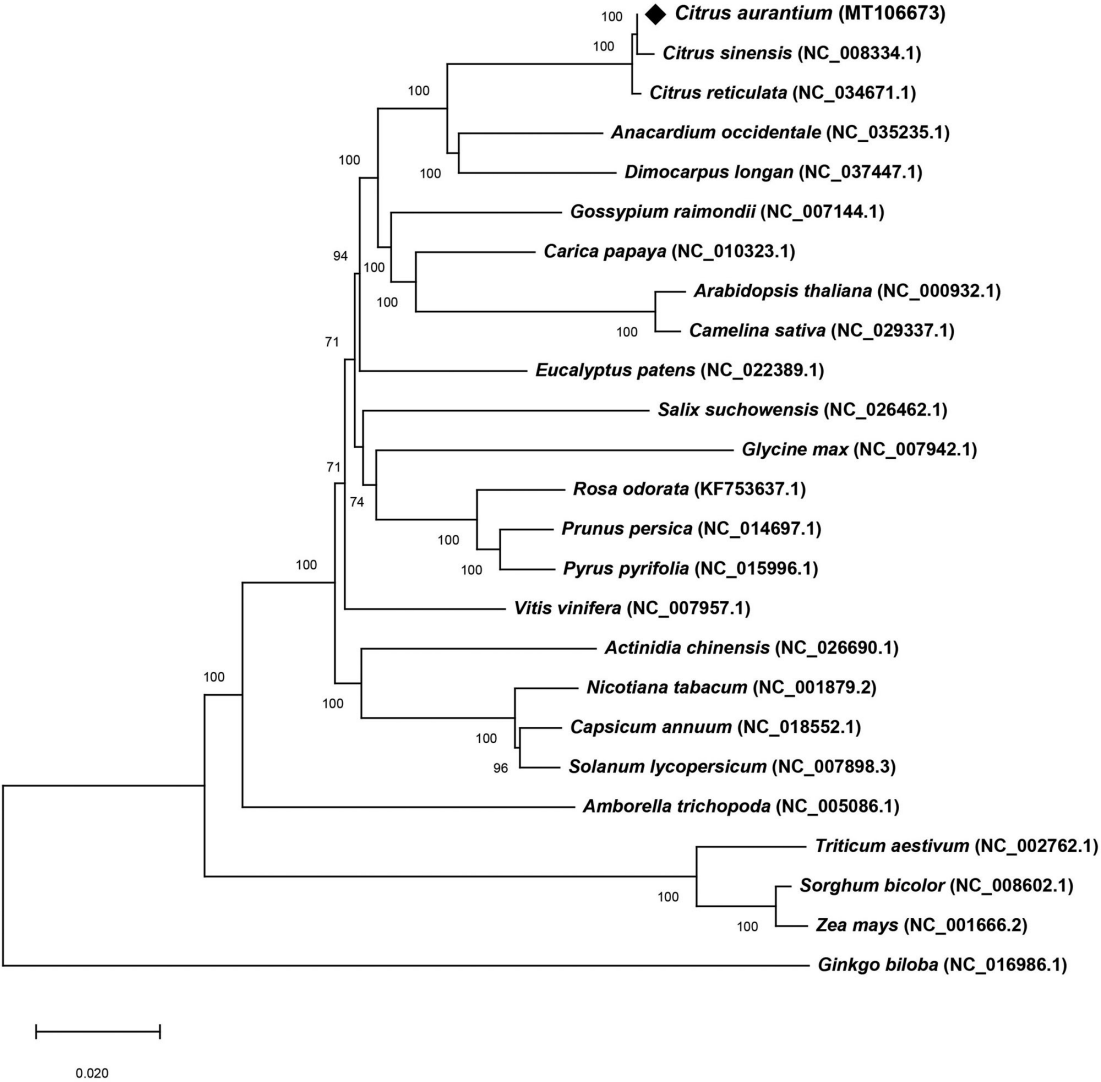
The NJ phylogenetic tree of 25 species based on 75 conserved protein-coding genes. Numbers in the nodes are bootstrap values from 1000 replicates. Accession numbers for tree reconstruction are listed right to their scientific names.

## Data Availability

The sequencing data that support the findings of this study are openly available in NCBI Short Read Archive (SRA) (accession number: SRR9127838). The assembled mitochondrial genome sequence of *Citrus aurantium* has been submitted to GenBank under the accession number: MT106672 (https://www.ncbi.nlm.nih.gov/nuccore/MT106672).
